# Dispensing pico to nanolitre of a natural hydrogel by laser-assisted bioprinting

**DOI:** 10.1186/1475-925X-10-19

**Published:** 2011-03-07

**Authors:** Martin Gruene, Claudia Unger, Lothar Koch, Andrea Deiwick, Boris Chichkov

**Affiliations:** 1 Laser Zentrum Hannover e.V., Hollerithallee 8, 30419 Hannover, Germany

## Abstract

**Background:**

Laser-assisted bioprinting of multi-cellular replicates in accordance with CAD blueprint may substantially improve our understandings of fundamental aspects of 3 D cell-cell and cell-matrix interactions *in vitro*. For predictable printing results, a profound knowledge about effects of different processing parameters is essential for realisation of 3 D cell models with well-defined cell densities.

**Methods:**

Time-resolved imaging of the hydrogel jet dynamics and quantitative assessment of the dependence of printed droplet diameter on the process characteristics were conducted.

**Results:**

The existence of a counterjet was visualised, proving the bubble collapsing theory for the jet formation. Furthermore, by adjusting the viscosity and height of the applied hydrogel layer in combination with different laser pulse energies, the printing of volumes in the range of 10 to 7000 picolitres was demonstrated. Additionally, the relationship between the viscosity and the layer thickness at different laser pulse energies on the printed droplet volume was identified.

**Conclusions:**

These findings are essential for the advancement of laser-assisted bioprinting by enabling predictable printing results and the integration of computational methods in the generation of 3 D multi-cellular constructs.

## Background

Bioprinting techniques are emerging as potential instruments for the multidisciplinary field of tissue engineering and regenerative medicine. The possibility to arrange multiple cell types in a computer-controlled 3 D manner may substantially improve our understanding about complex cell-cell and cell-environment interaction. Among all bioprinting techniques [1-3], laser-assisted bioprinting (LaBP) approaches based on laser-induced forward transfer were demonstrated to possess additional benefits: (i) tiny amounts of different hydrogels with a wide range of rheological characteristics can be printed in a controlled and precise way [4-8], which is important for the realisation of 3 D cell-hydrogel constructs mimicking various stiffnesses of native tissues; (ii) any desired cell amount ranging from single [9] to dozens of cells [10] can be printed without observable damage to pheno- and genotype [7,9-12]; and (iii) the printing speed (number of droplets per second) depends mainly on the pulse repetition rate of the applied laser. Printing speed of 5000 droplets per second was recently demonstrated [4], which enables fast generation of large cell constructs.

**Figure 1:**
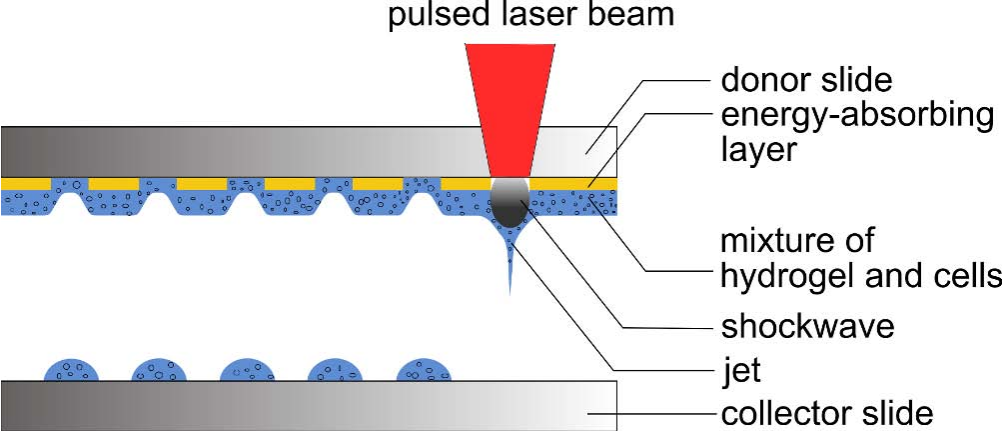
**Schematic Laser-assisted bioprinting setup**.

Already demonstrated biological applications reflect the flexibility of this laser printing technique, for instance: (1) generation and differentiation of 3 D stem cell grafts [13], which can be used as *in vitro *tissue models for the screening of drug effects; (2) assembly of cellular micro arrays of single [11] and multiple [14] cell types for systematic studies of fundamental aspects of cell-cell and cell-environment interaction; (3) computer-controlled seeding of 3 D scaffolds with multiple cell types [15]; and (4) *in vivo *bioprinting of nano-hydroxyapatite [16]. The principal laser-assisted bioprinting setup (see Figure 1) consists of a pulsed laser source and two positioning systems on which a donor-slide coated with an energy-absorbing material layer carrying the cell-hydrogel compound, and a collector-slide receiving the printed biological material are located. In brief, laser pulses are focussed through the donor-slide onto the gold layer which is evaporated locally at the focal point. This rapid energy deposition leads to the generation of a jet dynamic [17] resulting in the deposition of a tiny hydrogel volume on the collector-slide. Control of the printed volume is a key issue and great efforts have been made to understand the relationship between the printed volume and the processing parameters [5,6,8,18]. Providing a deeper understanding of this relationship is crucial in order to make the printed volume with embedded cells more predictable, and to enable theoretical simulation of cell-cell interaction, cell-extracellular matrix interaction and signalling pathways [12]. However, the whole jet generation process is not completely understood. Moreover, recent studies mainly used glycerol-based fluids to investigate the effects of the laser fluence and fluid properties on the droplet volume [5,8,18] instead of fluids based on fibrin-precursors, which are widely used for bioprinting of different cell types [4,7,13,15].

Therefore, in this study we present our experimental results concerning the relationship between the laser pulse energy and the rheological properties of a natural hydrogel consisting of alginate and blood plasma by means of time-resolved imaging and quantitative assessment of the droplet diameter.

## Methods

### Laser-assisted Bioprinting (LaBP)

A detailed description of the laser bioprinting setup has been previously published [7]. Briefly, to initiate the printing, a pulsed Nd:YAG laser (DIVA II, Thales, 1064 nm wavelength, 10 ns pulse duration, 20 Hz pulse repetition rate, beam quality M² < 1.1) was deployed. Laser pulse energies were varied by an attenuator and continuously monitored by an energy meter (Nova II and sensor 3A-P-V1, Ophir, Germany). Collector and donor glass slides (Resolab, Germany) were 26 × 26 × 1 mm in size and cleaned with acetone before usage. The bottom side of the donor glass slide was coated with a 60 nm gold layer using a plasma-enhanced sputter coater (Cressington 208HR, EO Service GmbH, Germany). The hydrogel layer was applied upon the gold layer by a blade coater. A 60 mm achromatic lens focused the laser beam through the donor-glass slide onto the gold layer.

The droplet deposition was controlled via computerized scanning setup consisting of three high speed translation stages (M-414.1PD and M413.3PD, Physik Instrumente GmbH, Germany). On the XY-translation stages, two mirrors and the Z-stage holding the focussing optics as well as the camera for process visualization were mounted. The stages were synchronized with laser pulses using a programmable computer-based real time system (Adwin-4L-T400, Jaeger Messtechnik, Germany) to ensure equidistant positioning of the laser spots. This automated CAM controlled stage setup allows single spot deposition and accurate positioning of a wide variety of patterns.

### Hydrogel

The utilized hydrogel consisted of alginate (low viscosity, brown algae) and ethylenediaminetetraacetic acid (EDTA) blood plasma. Chemicals were obtained from Sigma Aldrich, Germany, unless otherwise stated and porcine whole blood samples were obtained from a local abattoir. First 2 wt%, 4 wt% and 6 wt% of alginate were dissolved in 0.15 M NaCl-solution. These solutions were mixed 1:1 (v/v) with blood plasma resulting in 1 wt%, 2 wt% and 3 wt% alginate concentration. All the fluids show shear thinning flow behaviour which means that viscosity decreases with rate of shear. The material properties are shown in Table 1.

**Table 1:** Material characteristics of the hydrogels at 24°C

Alginate concentration	Density	Viscosity	Surface tension
(v/v)	(g/cm^3^)	(Pa·s)	(mN/m)
			
1%	1.017	0.022	46.7
2%	1.023	0.148	45.5
3%	1.028	0.431	45.3

The densities were acquired with a density meter DMA 38, Anton Paar, Austria. For viscosity measurements Fluids Spectrometer RFS II, Rheometrics Scientific, USA, was applied. Surface tension was measured by means of pendant drop method (OCA 40 micro, Dataphysics, Germany).

### Time-resolved imaging

The time-resolved imaging setup [17] consisted of a frequency-doubled Nd:YAG laser (Quanta-Ray DCR-11, 532 nm, 9.2 ns ± 0.5 ns (SD) pulse duration) for stroboscopic illumination and a single lens reflex (SLR) camera (Canon EOS 450D). Magnification of the images was realised by a five-fold microscope objective (Zeiss, Fluar, NA 0.25). The temporal delay between the laser responsible for printing and the illumination laser was set by two digital delay generators (SG 535, Stanford Research Systems, USA, and BME, SG05p, Bergmann Messgeraete Entwicklung, Germany). The delay was monitored by two fast-rising photo diodes (DET 10A/M, Thorlabs). With this setup, one frame per transfer could be captured. Therefore, at least 5 images per delay were taken to ensure reproducibility.

For the time-resolved imaging, the collector glass slide was removed. To prevent drying of the hydrogel layer, a humidity chamber was placed under the donor slide.

### Transferred volume

The determination of the transferred volume was accomplished by printing spot arrays, whereby every line corresponded to a certain energy level. At least 20 droplets per energy value were evaluated. To ensure reproducibility, three spot arrays for each chosen viscosity and layer height have been printed. Every spot array was printed from a freshly coated donor glass slide. Overall, this leads to 60 analysed droplets per each energy level, viscosity and layer height. Droplet diameters were automatically obtained by using the open source program ImageJ. Based on the average contact angle of 45.5°, the measured diameters were converted into volumes. The contact angle was investigated by dispensing small droplets on a cleaned glass substrate with a contact angle measuring device (OCA 40 micro, Dataphysics, Germany). Subsequently, the drying process was monitored and the contact angles were calculated with the help of the contact angle measuring device software.

Splashed volumes at higher energy have not been taken into account.

## Results

### Jet dynamics

**Figure 2:**
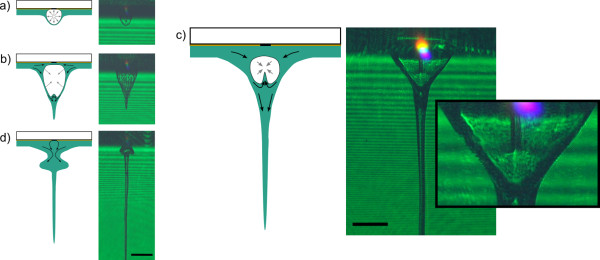
**Comparison of schematic and real jet formation images**. In the sketches, grey arrows indicate expansion or contraction of the vapour and black arrows mark the fluid flow inside the jet. For the time-resolved images hydrogel with the viscosity η = 0.1477 Pa·s was coated in 66 μm layer onto the donor slide. The black scale bars correspond to 200 μm. For images a), b) and d) laser pulse energy of 20 μJ was applied. The delays of the images were a) 1μs, b) 3 μs, c) 100 μs and d) 34 μs. To visualize the small counterjet higher energy levels were necessary. Therefore, applied energy in c) was 60 μJ.

Figure 2 shows the comparison of schematic and real jet formation images. For every delay, at least 5 images have been captured to ensure reproducibility. 1 μs after laser pulse impact, a small protrusion of the liquid layer is visible (Figure 2a). This protrusion grows and becomes elongated in shape after additional 2 μs (Figure 2b). At the tip, the jet forms. The existence a so-called counterjet was assumed for LaBP of liquids by Duocastella [8] and ourselves [17]. In Figure 2c) the counterjet is clearly visible. To uncover the small counterjet high energy levels for large size protrusion were necessary. The pulse energy was 60 μJ in comparison to 20 μJ for the other images. This led to temporal discontinuity of the images. Temporal delays were as follows: a) 1 μs, b) 3 μs, c) 100 μs and d) 34 μs. Figure 2d) shows a bulge structure around the jet which decreases over time.

### Viscosity effect

**Figure 3:**
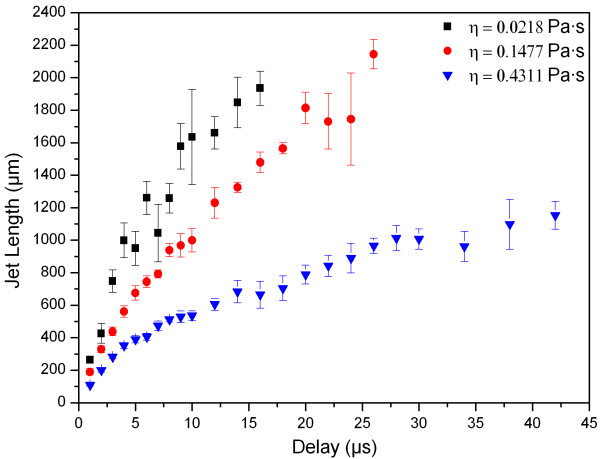
**Dependence of the jet evolution on viscosity**. Plot of the jet length dependence on the temporal delay. Five images per delay have been taken. The error bars mark the standard deviation.

**Figure 4:**
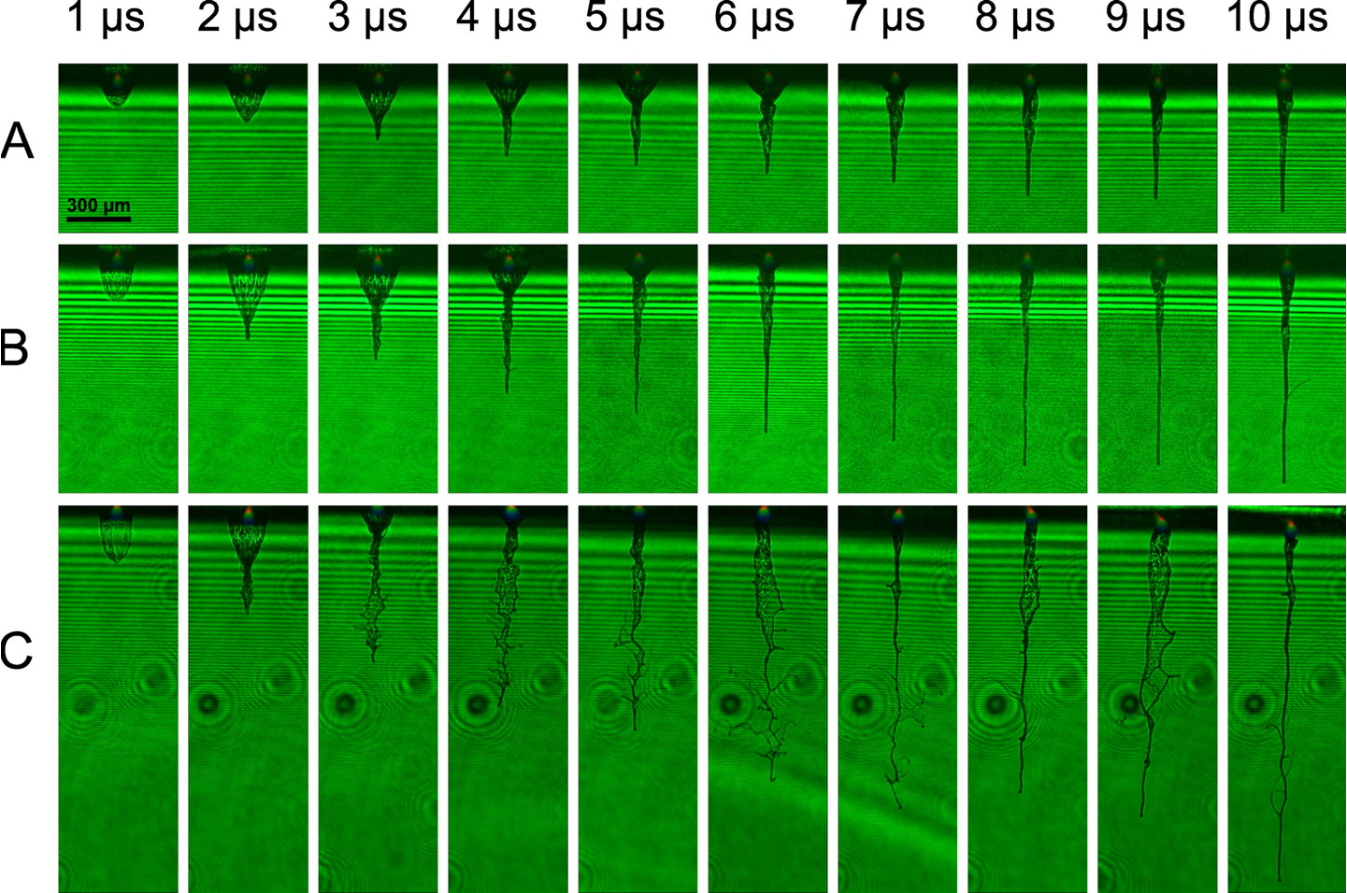
**Time-resolved imaging of jet formation depending on viscosity**. First 10 μs of the jet formation depending on the solution's viscosity whereas A corresponds to η = 0.4311 Pa·s, B to η = 0.1477 Pa·s and C to η = 0.0218 Pa·s. Black spherical disturbances in the background of C are due to condensed humidity on the microscope objective.

Figure 3 shows the jet length dependence on time for hydrogels with 0.0218 Pa·s, 0.1477 Pa·s and 0.4311 Pa·s viscosities. The laser energy has been kept constant at approximately 21 μJ during all the measurements. Five images per delay have been taken to ensure reproducibility. The error bars in the diagram represent the standard deviation. The plots for 0.0218 Pa·s and 0.1477 Pa·s viscosities end when the jets exceeded the image field of view. The plot for the lowest viscosity of η = 0.0218 Pa·s shows stronger inhomogeneities compared to other plots. As one can see in Figure 4C, the laser energy of 21 μJ is an upper limit for jetting behaviour for the 0.0218 Pa·s solution. In this case the jet appears very turbulent. Nevertheless, this laser pulse energy was chosen to observe jetting behaviour for all viscosities. For estimation of laminar or turbulent flow behaviour, the Reynolds number is usually applied. In our case, Reynolds numbers of the different viscosities are the following: R_e _(η = 0.0218 Pa·s) = 539, R_e _(η = 0.1477 Pa·s) = 51 and R_e _(η = 0.4311 Pa·s) = 13. For these calculations the jet's front velocity and jet diameter at 6 μs delay were used. Since the critical Reynolds number R_e, crit _for laser-assisted bioprinting of liquids has not been investigated so far, one cannot clearly distinguish between laminar and turbulent flows. However, these numbers support the time resolved images (Figure 4), where different flow behaviours of the hydrogels with η = 0.0218 Pa·s versus η = 0.1477 Pa·s and η = 0.4311 Pa·s are clearly visible. Further time-resolved imaging studies are necessary for verification of the critical Reynolds number during laser-assisted bioprinting of liquids. Figure 3 illustrates a distinct dependency of the jet evolution on the viscosity. During the first 5 μs of the jet formation process, the materials front velocity is very high. Later on the velocity is damped depending on the liquids viscosity.

Figure 4 clarifies the different velocities of the jet formation stages depending on the viscosity. At lower viscosity (C) the protrusion at the beginning is strongly pronounced since the hydrogel has lower resistance against expansion of the vapour. A flow inside the hydrogel is induced easier at low viscosity, leading to an earlier formation of the jet.

### Effect of layer thickness

**Figure 5:**
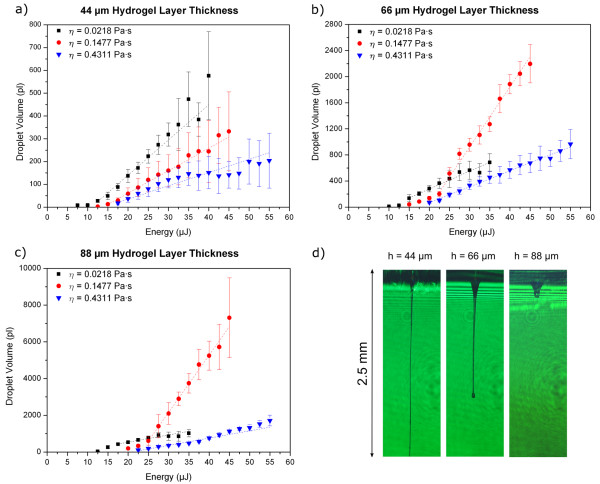
**Influence of layer thickness and viscosity on droplet volume**. Plots a) - c) show the dependence of the droplet volume on the laser energy and the initial hydrogel layer thickness on the donor slide. Dashed lines illustrate the linear trend of the data points and the error bars mark the standard deviation. For the time-resolved images in d) a solution with η = 0.4311 Pa·s was imaged at different layer heights. The temporal delay in all these images was 100 μs and the laser energy was kept constant at 17 μJ.

The influence of the layer thickness on the printed droplet volume was investigated at three different viscosities and over a wide range of laser energies. The investigated energy domains span from the minimum energy needed for material transfer and the energy level causing strong splashing of the droplets. In Figure 5 the relevant plots are demonstrated. From these graphs the following statements can be derived: (1) minimum transfer energies increase with rising viscosity and layer height, and (2) transferred volume increases with layer height at every viscosity. The most conspicuous dependency exhibits at η = 0.1477 Pa·s viscosity. At a layer thickness of 66 μm the maximal printed volume is about 6 times higher compared to a layer thickness of 44 μm, whereas for 0.0218 Pa·s and 0.4311 Pa·s viscosities the corresponding volume growth is 1.2 and 4.5 times, respectively. With increasing layer thickness this difference between the different viscosities becomes even more distinct.

**Figure 6:**
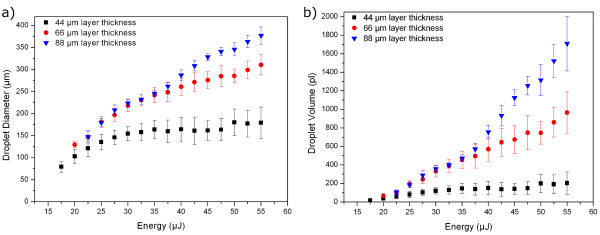
**Comparison of printed droplet diameter with droplet volume**. Progressions of the droplet diameter (a) and the droplet volume (b) depending on layer thickness and laser energy of a solution with η = 0.4311 Pa·s.

In Figure 6, the relationship between laser energy, droplet diameter and corresponding volume are clarified. As one can see in Figure 6b) only the droplet volume has nearly linear dependence on the laser pulse energy in contrast to Figure 6a) where the curve progression has more nonlinear character.

## Discussion

Laser-assisted arrangement of multiple cell types in accordance with a CAD blueprint provides a route for the realization of 3 D tissue constructs and the fabrication of niche-like environments resembling their native origin. We previously demonstrated the arrangement of cells in two [7] and three dimensions [13] on the micro-scale and without observable damage to their pheno- and genotype. However, the cells undergo mechanical shear forces during the jet formation by acceleration towards and impact on the collector slide. In order to predict these mechanical forces and their effects on the cells by means of numerical methods, the fundamentals of hydrogel flow inside the jet have to be understood to a greater extent. In previous studies, the mechanisms behind the jet formation process are discussed [5,8,17] and a first modelling attempt is presented [19]. The hydrogel transport initiated by LaBP proceeds in the following steps: first, the metal layer is irradiated by the nanosecond laser pulse. The laser light is absorbed by the electrons inside the solid and after only tens of picoseconds the atoms and electrons are in equilibrium state [20] which leads to strong heating of the material to the melting point. Subsequently the liquid material is vaporized by further nanosecond laser light absorption [21]. At low fluences the vapour is transparent for the laser light whereas at higher fluences the vapour is ionized by the laser irradiation and plasma is generated. Plasma shining is usually visible on the time-resolved images. Only at minimum transfer fluences plasma light cannot be detected. However, this is no evidence for absence of plasma. After the end of the laser pulse the vapour expands in all directions but resistance against expansion is lowest at the front because in this area the amount of hydrogel is small compared to the lateral region. Therefore the expanding vapour bubble possesses elongated shape (see Figure 2a). After the stretching of the hydrogel layer a flow inside of this layer is initiated. Due to inertia, surface tension and the bubble collapse the jet is formed and fed by the hydrogel flow (see Figure 2b). In studies focusing on cavitation bubble dynamics near free surfaces [22,23] an additional small jet penetrating the cavitation bubble is observed. Therefore, the existence of this so-called counterjet was assumed for LaBP of liquids by Duocastella [8] and ourselves [17]. In Figure 2c) the counterjet is clearly visible. The counterjet evolves due to bubble collapse and the high pressure region at the tip of the protrusion. In Figure 2d) the jet after collapse of the vapour is demonstrated. The lateral flows collide and the counterjet might be reflected on the donor glass slide, if its velocity is high enough to overcome viscous forces. Those colliding flows lead to a bulge formation. Over time, the bulge will decrease and move along the jet. This result can provide more precise boundary conditions for the computational fluid dynamic model and enables evaluation of critical flow forces onto cells, which are difficult to observe with present biological methods.

This work was carried out with an alginate-blood plasma hydrogel instead of a glycerol solution, as was primarily applied in other studies [5,8,18,24], due to three reasons. Firstly, alginic salts, in contrast to glycerol, are able to form an ionic network in the presence of bivalent cations (e.g. calcium), which is required for the generation of 3 D constructs. Alginate is biocompatible and has adjustable mechanical properties [25,26]. Blood plasma was chosen as the second hydrogel component due to simple withdrawal from the same cell donor and its coagulation ability (in the presence of calcium and/or thrombin). Secondly, hydrogels based on precursors of fibrin, in contrast to glycerol/water solutions, are widely used for biofabrication and tissue engineering applications [2,4,25,26]. Lastly, hydrogels with blood origin show shear-thinning flow behaviour whereas water/glycerol solutions have Newtonian properties. The resistance (viscosity) of a shear-thinning fluid decreases with higher rate of shear stress and increases with lower shear rates.

The cells inside such a hydrogel will undergo less shear forces during the acceleration phase of the jet, since the viscosity is decreased during this phase. The increasing viscosity during jet elongation, on the other hand, will decelerate the jet and thus reduce the impact forces onto the collector slide (Figure 3). Hence, with an adequate gap between the donor- and collector-slides these effects reduce shear stress on printed cells significantly compared to a Newtonian fluid.

Furthermore, we investigated the relationship between the laser pulse energy and gel layer characteristics (viscosity and layer height) of the applied hydrogel by means of time-resolved imaging and droplet size evaluation. Here, we demonstrate a nearly linear relationship between the laser pulse energy and the droplet volume. This relationship correlates with the results from other studies [8,24], working with a similar laser setup, metallic laser energy-absorbing layer and a water/glycerol solution with constant viscosity. Furthermore, we demonstrate that the droplet volume has no systematic dependence on the viscosity at different laser pulse energies. The droplet volume increased with rising viscosity until a maximum value at a viscosity of 0.1477 Pa·s is reached. Thereafter, the droplet volume decreased with further rising of the hydrogel's viscosity. This non-linear relationship between droplet volume and hydrogel viscosity correlates with the results of [5], who worked with a comparable setup and a water/glycerol solution, but without a metallic laser energy absorbing layer. Additionally, this effect was more pronounced when the height of the hydrogel layer was increased. Moreover, these results indicate that the maximum droplet volume at a specific viscosity depends on the height of the hydrogel layer. Based on these results two assumptions can be made:

(1) For every layer thickness a specific viscosity exists, where the printed droplet volume reaches its maximum.

(2) The specific viscosity, where the printed droplet volume reaches its maximum, reduces at a lower thickness of the hydrogel layer.

These observations are important for the development of analytical and computational approaches in order to clarify the relationship between material characteristics, laser pulse energy and the printed droplet volume. The theoretical understanding of this relationship may lead to a bioprinting approach that is capable of printing millions of femto to picolitre droplets of a crosslinkable hydrogel per second with any desired cell density.

## Conclusions

In order to make droplet deposition by a laser-assisted bioprinting approach more predictable, the fundamentals of the jet dynamics and the dependencies on laser pulse energy and fluid properties of a natural hydrogel were investigated by means of time-resolved imaging and quantitative assessment of the printed droplet diameter. These findings can be summarized as follows:

(1) Droplet volumes in the range of 10 to 7000 picolitres can be printed by adjusting the viscosity and thickness of the applied hydrogel layer in combination with the laser pulse energy.

(2) The existence of a counterjet has been proven, verifying the predicted bubble collapsing theory of the jet formation.

(3) Laser pulse energy and printed droplet volume have a nearly linear relationship at a constant viscosity and layer thickness in the energy regime examined.

(4) There is no systematic relationship between the viscosity, the layer thickness, and the printed droplet volume at different laser pulse energies.

These findings are important for the advancement of laser-assisted bioprinting due to enabling reliable and predictable volumes of transferred cell-hydrogels.

## Competing interests

The authors declare that they have no competing interests.

## Authors' contributions

MG and CU designed research, performed experiments, analyzed data, discussed the results and wrote the manuscript. LK and AD performed experiments and analyzed data. BC participated in the design of the study and coordination. All authors read and revised the manuscript.
